# Co-Transplantation of Human Neurotrophic Factor Secreting Cells and Adipose-Derived Stem Cells in Rat Model of Multiple Sclerosis

**DOI:** 10.22074/cellj.2018.4777

**Published:** 2018-01-01

**Authors:** Shahnaz Razavi Razavi, Nazem Ghasemi, Mohammad Mardani, Hossein Salehi

**Affiliations:** Department of Anatomical Sciences, School of Medicine, Isfahan University of Medical Sciences, Isfahan, Iran

**Keywords:** Adult Stem Cells, Lysophosphatidylcholines, Multiple Sclerosis, Myelinization, Nerve Growth Factors

## Abstract

**Objective:**

The presence of neurotrophic factors is critical for regeneration of neural lesions. Here, we transplanted combination of neurotrophic factor secreting cells (NTF-SCs) and human adipose derived stem cells (hADSCs) into a lysolecithin model of multiple sclerosis (MS) and determined the myelinization efficiency of these cells.

**Materials and Methods:**

In this experimental study, 50 adult rats were randomly divided into five groups: control, lysolecithin, vehicle, hADSCs transplantation and NTF-SCs/ hADSCs co-transplantation group. Focal demyelization was induced by lysolecithin injection into the spinal cord. In order to assess motor functions, all rats were scored weekly with a standard experimental autoimmune encephalomyelitis scoring scale before and after cell transplantation. Four weeks after cell transplantation, the extent of demyelination and remyelination were examined with Luxol Fast Blue (LFB) staining. Also, immunofluorescence method was used for evaluation of oligodendrocyte differentiation markers including; myelin basic protein (MBP) and Olig2 in the lesion area.

**Results:**

Histological study show somewhat remyelinzation in cell transplantation groups related to others. In addition, the immunofluorescence results indicated that the MBP and Olig2 positive labeled cells were significantly higher in co-cell transplantation group than hADSCs group (P<0.05). Also, outcome of motor functional test showed significant improvement function in cell transplantation groups, as compared to the others (P<0.01).

**Conclusion:**

Our results indicated that the remyelinization process in co-cell transplantation group was better than other groups. Thus, NTF-SCs/ hADSCs transplantation can be proper candidate for cell based therapy in neurodegenerative diseases, such as MS.

## Introduction

Demyelinating diseases, such as multiple sclerosis (MS), are characterized by the loss of oligodendrocytes, which predict demyelination and its attendant disability as well as neurological defects. MS is an inflammatory and a frequent demyelinating disease, involving central nervous system (CNS) that develops based on a complex genetic predisposition, such as the human leukocyte antigen locus on chromosome 6p21 (1) and environmental factors including exposure to infectious agents (Herpes virus type 6, Epstein-Barr virus and mycoplasma pneumonia) (2) together with vitamin deficiencies and smoking (3). 

These factors trigger a cascade of events in the immune system, which leads to neuronal, glial and axonal damages accompanied with nerve fiber demyelination. Pathogenetically, T cells, B cells, and their products are present in MS lesions and have a regulatory function in the progression of disease (4). CD8^+^ T cells mediated suppression of CD4^+^ T cells proliferation, promoted vascular permeability and activated oligodendrocyte death (5). Thus, an inflammatory response in CNS leads to multifocal demyelination, incomplete remyelination, scarring and astrogliosis in both white and gray matters. Current treatment options for MS are largely based on immunosuppressive agents, mostly interferon-ß (by reduced the production of proinflammatory cytokines) (6) and glatiramer acetate (by increased the expression of Foxp3 in CD4^+^, CD25^+^ T regulatory cells) (7). Since these treatments are partially effective in symptomatic alleviation and slowing down the progressive phase of MS, thus, several stem cell transplantation strategies have been proposed for the treatment of MS (8, 9). 

Previous study demonstrated that mesenchymal stem cells (MSCs) are able to secrete a variety of growth factors, strongly supporting the process of oligodendrocyte differentiation (8). 

Most of the studies focused on adipose-derived stem cells (ADSCs), because adipose tissue is an abundant and contain more stem cells than bone marrow (10). Moreover, ADSCs have potential to differentiate into multi lineage cells such as neural, glial (11-13), and neurotrophic factor secreting cells (NTF-SCSs) (14). ADSCs have additional properties including myelination master gene *Krox20* expression (15) and immunomodulatory effects that can alter the cytokine secretion profile of immune cells. ADSCs by secreting some growth factors including vascular endothelial growth factor (VEGF), basic fibroblast growth factor (bFGF), hepatocyte growth factor (HGF), neurotrophins (NT) such as brain-derived neurotrophic factor (BDNF), nerve growth factor (NGF), glial-derived neurotrophic factor (GDNF), NT-1 (16), and neuroregulins together with components of myelin sheath (17) may play an important role in remyelination and maintenance of the CNS functions. 

Previous study has shown that NTF-SCs secrete significant amounts of neurotrophic factors (NTF) when compared to ADSCs (14). So, cell therapy based on the transplantation of NTF-SCs derived from MSCs can be proper alternative in the treatment of neurodegenerative diseases. 

Recently, the successful experiment in animal models of neurodegenerative diseases has shown that NTF-SCs can play a pivotal role in impede various neurodegenerative processes (9). Consistent with these studies, the results of our previous study indicated that ADSCs could promote remyelination (18) and NTF-SCs derived from these cells are able to produce large amounts of NTFs (14). There are different methods for inducing MS model; we used local demyelinzation in spinal cord by lysolecithin. So, we can follow precisely myelin changes and fate of the injected cells in the site of lesion after transplantation. Thus, NTF- SCs can be transplanted safely into MS lesions and thereby serve as vehicles for delivering NTFs in order to promote stem cell differentiation. Therefore, in accordance to all of the aforementioned, we evaluated the effects of *in vivo* co-transplantation of NTF-SCs/ hADSCs in demyelinated spinal cord rat as a model of MS.

## Materials and Methods

In this experimental study, all of the used materials were prepared from Sigma-Aldrich, USA. Meanwhile, all methods were certified by the Ethics Committee of Isfahan University of Medical Sciences. After receiving informed consent of female donors, hADSCs were obtained from human abdominal fat and cultured as previously explained (19). 

Briefly, the fat tissues were washed twice with phosphate- buffer saline (PBS) in order to eliminate contaminating debris and then enzymatic degradation was performed by 0.075% collagenase type I in a 37°C humidified incubator for 30 minutes. After neutralizing enzyme activity with Dulbecco’s Modified Eagles Medium (DMEM, Gibco, UK) containing 10% fetal bovine serum (FBS, Gibco, UK), the suspended cells were centrifuged for 10 minutes at 1200 rpm and the obtained cellular pellet was resolved in basic medium supplemented with 1% penicillin/ streptomycin solution. 

The primary cells culture was performed for 4-5 days at standard condition and when the cell confluency reached to nearly 80%, the cells were passaged with 0.25% trypsin and 0.02% ethylenediaminetetraacetic acid (EDTA). 

### Characterization of human adipose derived stem cells

Flow cytometer technique was performed for characterization of hADSCs using FITC or phycoerythrin conjugated antibodies against CD90, CD44, CD195, CD34, CD14, and CD45 (Chemicon, CA, USA) for 30 minutes. Meanwhile, for isotype control, nonspecific FITC-conjugated IgG was substituted for the primary antibodies. 

### Induction of human adipose deriverd stem cells into neurotrophic factor-secreting cells

The induction of hADSCs into NTF-SCs was carried out according to the previous study (20). HADSCs (1×10^6^ cells) were cultured in a pre-differentiation medium containing of DMEM/F12 (Gibco, UK) (SPN, L-glutamine) supplemented with 20 ng/ml human bFGF (hbFGF, Gibco, UK), 20 ng/ml human epidermal growth factor (hEGF) and N2 supplement for 3 days. Then, terminal differentiation was induced in DMEM/F12 (SPN, L-glutamine) supplemented with 1 mM dibutyryl cyclic AMP (dbcAMP), 0.5 mM isobutylmethylxanthine (IBMX), 5 ng/ml human platelet derived growth factor (PDGF), 50 ng/ml human neuregulin 1-b1/HRG1-b1 EGF domain and 20 ng/ml hbFGF for 3 days. 

### 3-[4, 5-dimethylthiazol-2-yl]-2,5-diphenyl tetrazolium bromide (MTT) Assay 

The cell viability and proliferation of NTF-SCs was examined using MTT assay. The stock solution of MTT (5 mg/ml) was added to the culture medium at a dilution of 1:10 and the plates were incubated at 37°C for 4 hours. Then, medium was aspirated and 200 µl of dimethyl sulfoxide (DMSO) was added to each well and the absorbance of the solution in each well was determined by using a microplate reader (Hiperion MPR 4+, Germany) at 540 nm. 

### Cell labeling with PKH26 and Hoechst 

NTF-SCs/ hADSCs labeling with PKH26 and hoechst was performed according to manufacturer’s guideline. Briefly, 1×10^6^ cell/ml concentration was prepared and PKH26 was added, followed by incubation for 1-5 minutes. Next, 1% bovine serum albumin (BSA) was used to stop labeling. In the following step, cell washing was done by DMEM/ F12 medium and an aliquot of these cells was checked by fluorescent microscopy (Olympus BX51, Japan) to determine the staining efficiency. In addition, a few labeled cells were cultured to confirm cell viability. 

In order to hADSCs labeling with Hoechst, DMEM/ F12 medium contain Hoechst (10 µg/ml) was added to 1×10^6^ cells of hADSCs and then incubated for 30-60 minutes. After medium aspiration, the samples were washed twice with DMEM/F12 in order to eliminate additional dye. Finally, staining efficiency was check by fluorescent microscopy. 

### Rat spinal cord demyelinization

Fifty male Wistar rats, weighing 200-250 g, were prepared from Pasteur Institute (Tehran, Iran) and communally housed on a 12-hour light/dark cycles with free access to water and standard dry diet. All animal experiments were approved by the Animal Ethics Committee of Isfahan University of Medical Sciences. 

In this study, random sampling method was used and the rats were divided into following groups: control (only laminectomy; n=10), lysolecithin (laminectomy and demyelination with lysolecithin, n=10), vehicle control (laminectomy, demyelination and medium injection instead of cells transplantation, n=10), hADSCs transplantation (laminectomy, demyelination and hoechst-labeled hADSCs transplantation, n=10) and NTF-SCs/hADSCs co-transplantation (laminectomy, demyelination and PKH- labeled NTF-SCs/hoechst-labeled hADSCs transplantation, n=10). After anesthetizing animals, laminectomy and dura exposing were carried out at the level of T9/11 vertebra. In following step, through a glassy micropipette, 2 µl solution of 1% lysolecithin was injected slowly into the lateral column of the spinal cord. In order to avoid backflow of the lysolecithin, micropipette was left in injection site for an additional 2 minutes. After suturing, 15 mg/kg Gentamycin (Hakim Pharmaceutical, Iran) and 5 ml lactated ringers’ solution (Hakim Pharmaceutical, Iran) were given to each animal and they were kept on a heating pad until fully awake, followed by housing them in standard rat cages.

### Cells transplantation into the lysolecithin-treated spinal cord 

According to the previous studies, NTF-SCs/hADSCs transplantation was done one week after including demyelination. In order to suppress the immune system of rats, 15 mg/kg cyclosporine A (Sand immune, Novartis Pharmaceuticals, USA) was intraperitoneally (I.P) administered per day (from a day before transplantation until the end of the study). After induction of anesthesia, laminectomy area was re-exposed and 1×10^6^ hADSCs, 5×10^5^ NTF-SCs with 5×10^5^ hADSCs and 10µl medium were injected by a glassy micropipette on the level of demyelization lesion in hADSCs, NTF-SCs hADSCs transplantation and vehicle groups, respectively (18, 21).

### Immunofluorescence staining 

At the endpoint of experiment, the rats were anesthetized and fixing process was performed through transcardially perfusion methods with ice-cold PBS and 4% paraformaldehyde (PFA) in PBS (pH=7.4). Rat spinal cord was removed and postfixed in the same fixative at 4°C overnight. Then, the sample was cryoprotected by 30% sucrose (Sigma-Aldrich, USA) in PBS for 48-72 hours. Subsequently, serial frozen sections (10 µ thick) of the spinal cords were prepared using a microtome cryostat. In order to evaluate the presence of myelin forming cells in transplantation area, immunofluorescence technique was done with primary antibodies include mouse monoclonal anti-MBP (1:1000), mouse monoclonal anti-Olig2 (1:1000) and Goat anti-mouse FITC (1:2000, all purchased from Abcam, UK) as secondary antibody. Finally, after labeling the cell nucleus using 4', 6-Diamidino2- Phenylindole, Dilactate (DAPI) cells were observed using a fluorescence microscope, and immunopositive cells was counted in a minimum total of 200 cells per slide. Meanwhile, all immunofluorescence studies were repeated at least twice. 

### Myelin staining 

Myelin content was determined by Luxol Fast Blue (LFB) staining. The thin sections (10 µ thick) were stained overnight in LFB solution at 56°C and counterstained with cresyl violet solution for 30-40 seconds. After washing with PBS and differentiation with 95% ethyl alcohol, the samples were assessed with invert microscopy (Nikon, Japan). 

### Statistical analysis

Statistical analysis was performed by independent sample t test and one-way analysis of variance (ANOVA). Data was presented as mean ± SEM and values of P<0.05 was considered to be statistically significant. 

## Results

### Human adipose derived stem cells/neurotrophic factor secreting cells characterization 

**Fig.1:**
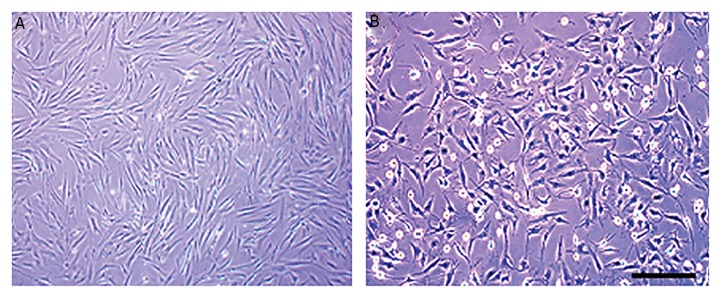
Phase contrast images of cell morphology. A. Morphological changes were observed in human adipose-derived stem cells (hADSCs) during neurotrophic factor secreting cells (NTFSCs) differentiation. Cultured hADSCs in the third passage and B. hADSCs induced to NTF-SCSs differentiation at the end of differentiation process (scale bars=150 µm).

HADSCs in primary culture exhibit fibroblast-like morphology (Fig .1A). In addition, these cells were CD44, CD90, and CD105-positive, but negative for CD14, CD34, and CD45 (hematopoietic markers, data has not been shown). After differentiation of hADSCs into NTF-SCs, differentiated cells display a satellite-like morphology (Fig .1B). Moreover, in our pervious study using different methods, the capability and level of neurotrophic factor secretion were demonstrated in differentiated cells (20).

### Cell viability assessment

We examined the survival and proliferative potential of the induced hADSCs using MTT assay. The mean absorbance value of the NTF-SCs (0.74 ± 0.20) was significantly increased as compared to hADSCs (0.49 ± 0.10, P<0.05). Therefore, the induced NTF-SCs not only can be survived but also propagated, in the presence of induction medium. 

### Histological study of cell transplantation 

**Fig.2:**
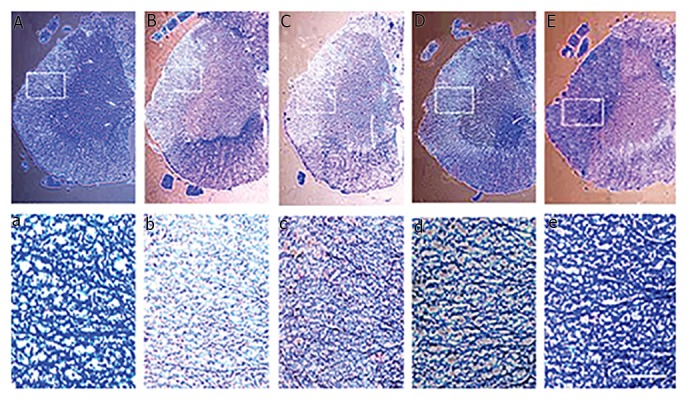
Luxol fast blue and cresyl violet staining of the spinal cord (lateral funiculus) at four weeks after (NTF-SCs)/hADSCs transplantation. A, a. Normal myelin tissue in control group, slight remyelination observed in B, b. Lysolecithin, C, c. Control vehicle groups, moderate remyelination observed in D, d. hADSCs transplantation group, and E, e. hADSCs/NTF-SCs transplantation group remyelination was nearly to the control group [scale bars=200 µm in (A-E) and 100 µm in the box (a-e)]. hADSCs; human adipose-derived stem cells and NTF-SCs; Neurotrophic factor secreting cells.

Four weeks after cell transplantation, rats were sacrificed, and spinal cord tissues were examined histologically. Injection of lysolecithin into the lateral funiculus of the spinal cord consistently resulted in a focally demyelinated zone as shown by LFB/cresyl violet staining of frozen sections from the lesion. A moderately remyelinization was obtained in the hADSCs transplantation region, while recovery of myelination in co-transplantation group was close to the control group (Fig .2). 

### Immunofluorescence study of cell transplantation

**Fig.3:**
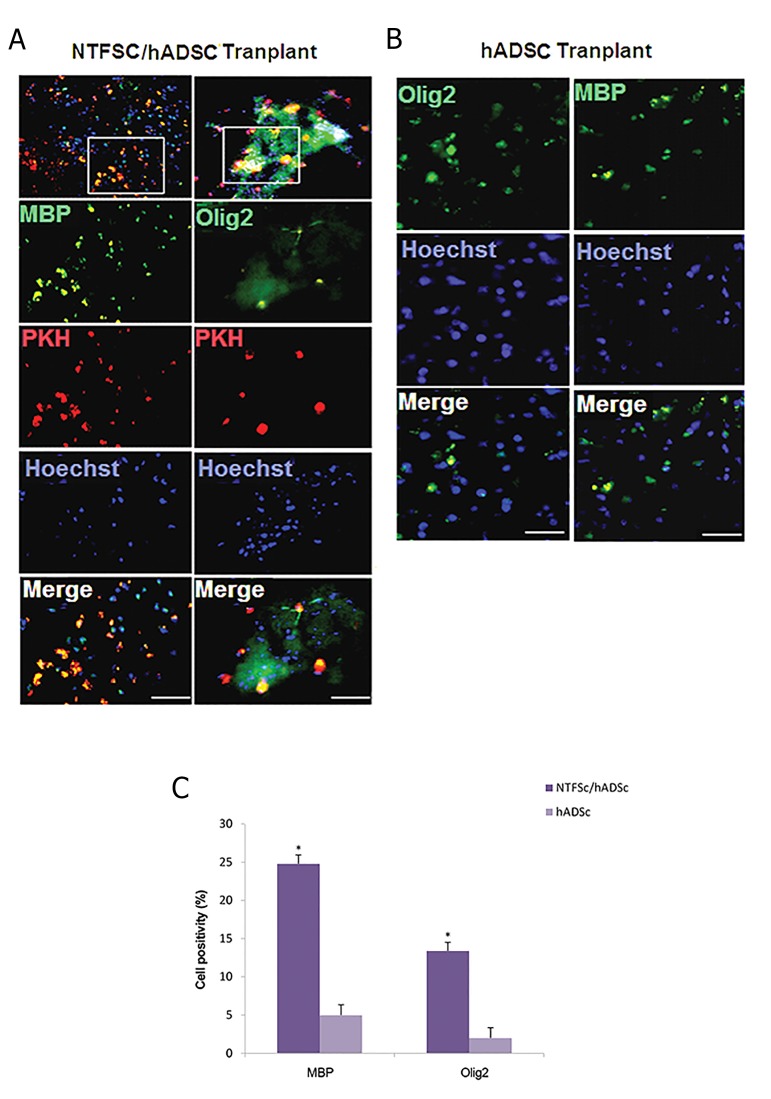
Longitudinal sections of lysolecithin lesions and immunohistochemistry staining four weeks after cell transplantation. A, B. NTF-SCs were pre-labeled with PKH26 (red) and hADSCs were pre-labeled with Hoechst (blue) to show all the cells, and C. In NTF-SCs/ hADSCs transplantationgroup the presence of Olig2 and MBP positive cells were significantly higher as compared to hADSCs transplantation group (*; P<0.05) (scalebars=100 µm).

Immunofluorescence staining for oligodendrocyte specific markers was used to identify the phenotype of oligodendrocyte cells in the lysolecithin lesions. The results were depicted in hADSCs/NTF-SCs transplantation group, 13.4 ± 1.11% of transplanted cells was positive for Olig2, a marker for immature oligodendrocyte, and 24.8 ± 1.14% of them was positive for MBP, a marker for mature oligodendrocyte (Fig .3A). Furthermore, in hADSCs transplantation group, 5 ± 1.34% of the transplanted cells was positive for MBP and 2 ± 1.3% of them was positive for Olig2 (Fig .3B), which was significantly lower than cotransplantation group (P<0.05, Fig .3C).

### Assessment of motor functions 

**Fig.4:**
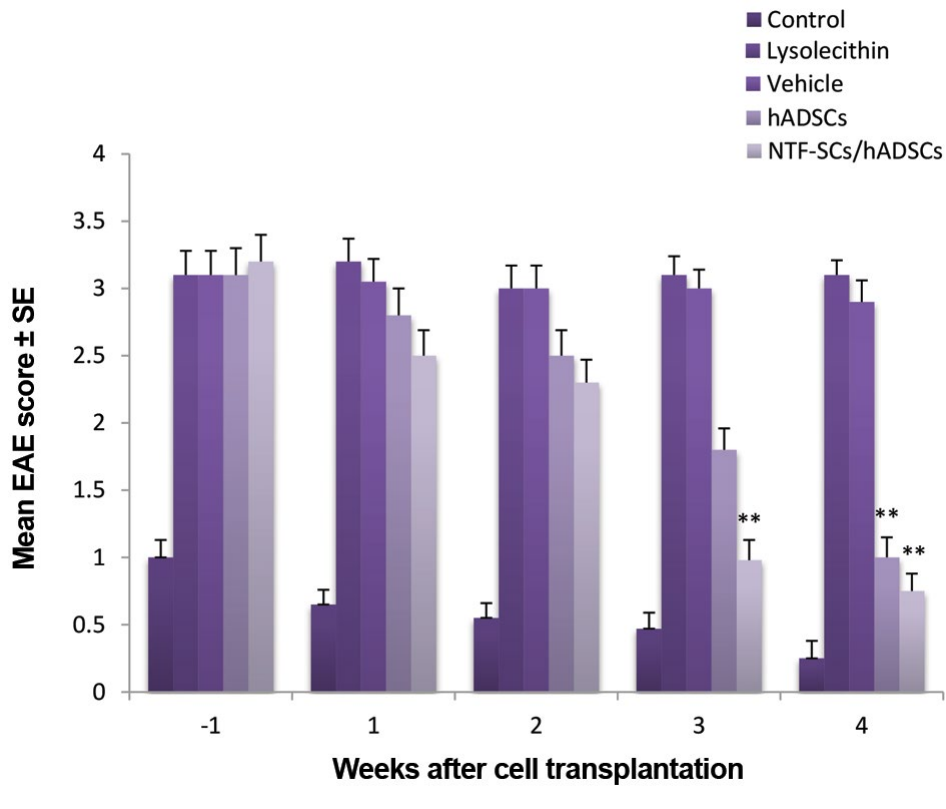
Motor functional recovery assessment from one week before, to 4 weeks after cell transplantation by experimental autoimmune encephalomyelitis (EAE) scoring scale. In hADSCs group a significant decrease of functional score was obtained relative to control and vehicle groups four weeks after cell transplantation (**; P<0.01). While, a significant recovery of motor activity was determined in NTF-SCs/hADSCs transplantation group, three and four weeks after cell transplantation, as compared to others (**; P<0.01). hADSCs; Human adipose-derived stem cells and NTF-SCs; Neurotrophic factor secreting cells.

The disease course of all rats was evaluated with the classical EAE-scoring scale as described previous (22). All animals were scored weekly from the since lysolecithin lesion to four weeks after cell transplantation. In the cell transplantation groups, all rats received daily cyclosporine A from one day before cell transplantation. Cyclosporine is effective in preventing cell rejection. Moreover, it has no effect on the clinical and pathological course of MS model (23). The results of clinical scores demonstrated that at three and four weeks after cell transplantation, the clinical signs of spinal cord injury were significantly alleviated in co-transplantation group as compared to other groups (P<0.01). Additionally, in hADSCs group a significant decrease of functional score was obtained relative to control and vehicle groups after four weeks of transplantation (P<0.01). However, the functional score of hADSCs was higher than co-cell transplantation group (Fig .4). 

## Discussion

MS is young adults’ onset neurodegenerative diseases leading to progressive myelin destruction within the CNS which is accompanied with a physical or cognitive disability. Multifocal regions of inflammation into CNS are the primary cause of damage in MS (4). 

The current disease modifying treatment for MS is based on the use of immunomodulatory and immune suppressive strategies (24). The main mechanism of action of these agents is not clear; nevertheless, several potential pathways have been proposed. Among these mechanisms, the release of transforming growth factor (TGF)-beta and Th2 cytokine production, ameliorate myelination (25), inhibiting B and T cell migration through the blood brain barrier (26), increasing cytokine levels such as interleukin (IL)-10, tumor necrosis factor-alpha, and IL-4 (27) and decrease in matrix metalloproteinase activity (28) may be significant. 

These treatments are just somehow effective in attenuating the MS progression. Thus, a more effective treatment strategy is needed which can prevent disease progression. Stem cells are a promising strategy for the treatment of neurodegenerative diseases due to their immunomodulatory and neuroprotective potential effects. For example, Karussis et al. (29) reported that MSCs transplantation in patients with neurodegenerative diseases is a reliable method due to immunomodulatory effects of the transplanted cells. In addition, another study suggested that immunoregulatory and trophic effects of MSCs may have therapeutic value in stem cell therapy (30). Our previous studies have demonstrated that hADSCs can be persuaded *in vitro* to produce and release a number of trophic factors (14, 20). Moreover, hADSCs transplantation in the rat model of MS showed that these cells participate to remyelination by differentiating into mature oligodendrocyte and activating oligodendrocyte progenitor cells (18). Hence, administration of these cells can ameliorate neurodegenerative diseases.

In this study, hADSCs were differentiated into NTF secreting cells and then co-transplanted in the rat model of MS disease. Histological analysis demonstrated that transplantation of hADSCs with or without NTF-SCSs can reduce the areas of demyelination and enhance remyelination. One hypothesis for this event may be that NTF-SCs secreted higher levels of NT factors which can support the survival and proliferation of hADSCs and promote oligodendrocyte differentiation as well as remyelination process which is consistent with several recent studies (31-34). 

Our behavioral results further confirmed a significant improvement in motor functional recovery based on EAE scoring scale in cell transplantation groups. More improvement in co-cell transplantation might be explained by the fact that NTF-SCs have a key role in releasing NTF, reduction of apoptosis and supporting the proliferation of the exogenous cells that is consistent with modulation of the immune response and enhancement of oligodendrocytes differentiation which promoting myelin repair. These data supported the results of previous studies that suggested the delivery of NTFs, such as bFGF and BDNF induced a beneficial effect of clinical and pathological scores with an increase of mature oligodendrocytes and their progenitors in an EAE model of MS (34, 35). However, NT factors have short half-life and when delivered peripherally their efficacy in the CNS, it is reduced due to the blood- brain barrier. So NTF-SCs could be an ideal vehicle for delivering NTFs into the CNS lesions.

## Conclusion

The results of this study show that the transplantation of NTF-SCs along hADSCs in lysolecithin lesion through NTF delivery can induce differentiation of exogenous hADSCs into oligodendrocyte cells and improve remyelinization that lead to develop motor function. Hence, hADSCs/NTF-SCs co-transplantation may be an ideal candidate for cell based therapy in neurodegenerative diseases, such as MS. 
